# Megakaryocyte NLRP3 hyperactivation induces mild anemia and potentiates inflammatory response in mice

**DOI:** 10.3389/fimmu.2023.1226196

**Published:** 2023-08-09

**Authors:** Joshua H. Bourne, Joana Campos, Sophie J. Hopkin, Katharine Whitworth, James Palis, Yotis A. Senis, Julie Rayes, Asif J. Iqbal, Alexander Brill

**Affiliations:** ^1^ Institute of Cardiovascular Sciences, College of Medical and Dental Sciences, University of Birmingham, Birmingham, United Kingdom; ^2^ Centre for Inflammatory Diseases, Department of Medicine at Monash Health, School of Clinical Sciences, Monash Medical Centre, Monash University, Clayton, VIC, Australia; ^3^ Department of Pediatrics, University of Rochester Medical Center, Rochester, NY, United States; ^4^ Etablissement Français du Sang, Inserm Institut National de la Santé et de la Recherche Médicale (INSERM), Unité Mixte de Recherche (UMR)-S1255 Strasbourg, Université de Strasbourg, Strasbourg, France

**Keywords:** NLRP3, platelets, inflammasome, zymosan-induced peritonitis, erythropoiesis

## Abstract

**Background:**

The NOD-, LRR-, and pyrin domain-containing protein 3 (NLRP3) inflammasome has been described in both immune cells and platelets, but its role in the megakaryocyte (MK) lineage remains elusive.

**Objective:**

The aim of this study was to explore the role of NLRP3 inflammasome in megakaryocytes and platelets.

**Methods:**

We generated *Nlrp3* ^A350V/+^/*Gp1ba-*Cre^KI/+^ mice carrying a mutation genetically similar to the one observed in human Muckle–Wells syndrome, which leads to hyperactivity of NLRP3 specifically in MK and platelets.

**Results:**

Platelets from the mutant mice expressed elevated levels of both precursor and active form of caspase-1, suggesting hyperactivity of NLRP3 inflammasome. *Nlrp3* ^A350V/+^/*Gp1ba-*Cre^KI/+^ mice developed normally and had normal platelet counts. Expression of major platelet receptors, platelet aggregation, platelet deposition on collagen under shear, and deep vein thrombosis were unchanged. *Nlrp3* ^A350V/+^/*Gp1ba-*Cre^KI/+^ mice had mild anemia, reduced Ter119^+^ cells in the bone marrow, and splenomegaly. A mild increase in MK TGF-β1 might be involved in the anemic phenotype. Intraperitoneal injection of zymosan in *Nlrp3* ^A350V/+^/*Gp1ba-*Cre^KI/+^ mice induced increased neutrophil egression and elevated levels of a set of proinflammatory cytokines, alongside IL-10 and G-CSF, in the peritoneal fluid as compared with control animals.

**Conclusion:**

MK/platelet NLRP3 inflammasome promotes the acute inflammatory response and its hyperactivation in mice leads to mild anemia and increased extramedullary erythropoiesis.

## 1 Introduction

Platelets, once thought of as bystanders during inflammation, are now accepted as mediators of the inflammatory response. Platelets have recently been shown to contain all components of the NOD-, LRR-, and pyrin domain-containing protein 3 (NLRP3) inflammasome, a molecular complex leading to activation of caspase-1, which, in turn, activates interleukins (IL)-1β and 18 (1). NLRP3 inflammasome assembly was initially described in immune cells following activation by proinflammatory stimuli, damage- and pathogen-associated molecular pattern (DAMP and PAMP, respectively). In platelets, inflammasome assembly has been associated with sickle cell disease, Crohn’s disease, and acute coronary syndrome (2–4). Exposure to Dengue virus results in reactive oxygen species (ROS)-triggered NLRP3 inflammasome assembly in platelets leading to the synthesis of IL-1β and its secretion in association with microparticles (1). This, in turn, increases vascular permeability, thus contributing to the inflammatory process. We have recently reported platelets as one of the major sources of caspase-1 in murine venous thrombi, which promoted thrombosis in conjunction with neutrophil extracellular traps (5).

A constitutive *Nlrp3* knockout mouse model has provided important insights into the pathophysiological functions of the NLRP3 inflammasome but has failed to delineate the lineage-specific contributions to the inflammatory response. Notably, the global *Nlrp3* knockout mouse was protected from sepsis and NLRP3 expression was shown to be essential in ATP-driven caspase-1 activity, and subsequent IL-1β secretion (6–8).

Hyperactivation of the NLRP3 inflammasome is observed in patients with Muckle–Wells syndrome, characterized by a A350V mutation of the *Nlrp3* gene. Here, we characterize a novel mouse, *Nlrp3* ^A350V/+^/*Gp1ba-*Cre^KI/+^, to assess the role of the NLRP3 specifically in the megakaryocyte (MK)/platelet lineage. We demonstrate that NLRP3 hyperactivity results in elevated amounts of caspase-1 in platelets, whereas the levels of systemic IL-1β remain unchanged. The mutation does not alter platelet function, thrombus formation *in vitro*, and venous thrombosis *in vivo*, but results in unexpected mild anemia, splenomegaly, and robust increase in neutrophil recruitment and inflammatory cytokines in the peritoneal lavage fluid (PLF) following zymosan-induced peritonitis. Thus, MK/platelet NLRP3 inflammasome is involved in erythropoiesis and acute inflammatory response.

## 2 Methods

### 2.1 Mice

*Nlrp3^A350VneoR^* knock-in mice of 10–14 weeks old, on C57BL/6 background, in which a *loxP*-flanked neomycin resistance cassette is inserted in reverse orientation into intron 2 of the *Nlrp3* gene, were purchased from Jackson Lab (strain 017969). These mice have a missense mutation, A350V, in the exon 3 on the *Nlrp3* gene, which resembles the mutation observed in human Muckle–Wells syndrome. *Nlrp3^A350VneoR^* knock-in mice were crossed to *Gp1ba-*Cre^KI/+^ mice (9) to generate mutation specific for MK/platelet lineage. The resulting *Nlrp3* ^A350V/+^/*Gp1ba-*Cre^KI/+^ mice were used in the experiments with *Nlrp3* ^A350V/+^/*Gp1ba-*Cre^+/+^ and *Nlrp3* ^+/+^/*Gp1ba-*Cre^KI/+^ littermates serving as a control. As *Nlrp3* and *Gp1ba* are both located close (~10 MBs) on chromosome 11, breeding efficacy of homozygous animals was very low and therefore we used heterozygous mice. Both male and female mice aged 8–14 weeks were used. All experiments were performed in accordance with UK law (Animal Scientific Procedures Act 1986), with approval of the local ethics committee and UK Home Office.

### 2.2 Platelet isolation and aggregation

Blood was drawn from the inferior vena cava (IVC) of anesthetized mice to 10% anti-coagulant citrate dextrose (ACD). Blood counts were performed using an ABX Pentra 80 automated hematology analyzer (HORIBA). Platelets were isolated by centrifugation using Modified Tyrode’s Buffer (pH 7.4) and washed in the presence of prostacyclin (1 µg/ml). Platelet aggregation at 2 × 10^8^/ml was assessed by light transmission aggregometry (ChronoLog, USA) in glass vials at 37°C, stirring at 1,200 rpm for 6 min, in the absence or presence of an agonist.

### 2.3 Flow adhesion models

Whole blood was perfused at arterial shear rate (1,000 s^−1^) as described (10). In brief, whole blood was perfused over Horm collagen (coated at 100 µg/ml) through a µ-Slide VI 0.1. Blood was treated with 1 µM DiOC6 (ThermoFisher) and 40 µM PPACK. Images were acquired simultaneously of two fields with Z-stacks using an EVOS M5000 Imaging system (ThermoFisher) by fluorescence (20× lens). Platelet deposition was calculated as percentage of surface coverage using FIJI v2.1.

### 2.4 Deep vein thrombosis model

Deep vein thrombosis (DVT) was modeled by IVC stenosis as described (11). In brief, mouse was placed in the supine position and anesthetized with isoflurane–oxygen mixture, and laparotomy was performed through midline incision. Guts were exteriorized and IVC was gently separated from aorta. The IVC was closed over a spacer (30G needle) with a 7-0 prolene suture, after which the spacer was removed. This results in ~90% closure of the IVC lumen (12). All visible side branches were ligated. Mice were euthanized and thrombosis checked in 48 h.

### 2.5 Bone marrow immunohistochemistry

The femur and tibia were harvested and cleaned from surrounding tissues. One side of the bone was cut using sterile scissors, before fixation in 4% formalin for 24 h. Bones were then decalcified in EDTA (500 mM) for 72 h, or until rigidity is lost, and embedded in paraffin. Tissue was sectioned to 8 µm, deparaffinized with xylene, and rehydrated before antigen retrieval in antigen unmasking solution (Vector Labs, UK) and blocking using REAL peroxidase blocking solution (DAKO, UK). Sections were stained using a Hematoxylin & Eosin (H&E) kit (abcam, UK). Tissue was dehydrated and mounted with VECTASHIELD anti-fade mounting medium (Vector Labs, UK). Tissue was imaged using a Zeiss AxioScan.Z1 slide scanner.

### 2.6 Spleen immunofluorescence

Spleens were harvested, weighed, and halved (coronally), before embedding in optical cutting temperature (OCT) and snap freezing in liquid nitrogen. Spleens were then cyrosectioned to 6 µm, mounted on slides, and stored at −80°C. Sections were fixed in ice-cold acetone before blocking in 3% BSA and 5% donkey serum in TBS for 1 h. Sections were incubated with primary antibodies Ter119 (Ter119-AF488, BioLegend), CD3e (145-2C11-Hamster, eBioScience), and CD19 (eBio1D3-Rat, eBioScience) at 4°C overnight. Sections were TBS-T-washed, before incubation with secondary antibodies for 1 h at room temperature, and then washed. Autofluorescence was quenched by ammonium chloride (50 mM), and tissue was flat mounted with VECTASHIELD anti-fade mounting medium (Vector Labs, UK). Organs were imaged using a Zeiss AxioScan.Z1 slide scanner, and images were compiled using FIJI v2.1.

### 2.7 Flow cytometry

Bone marrow (BM) was isolated from the femur and tibia of mice by centrifugation, as described (13), and filtered through a 70-µm filter unit. The spleen was homogenized through a Falcon 70-µm cell strainer using the flat end of a 1-ml syringe, before final filtration through a 70-µm filter unit. Whole blood was drawn into 10% ACD, and diluted 1:10 in Tyrode’s Buffer. Cells were incubated in the presence of conjugated antibodies for 20 min on ice: CD41 (MWReg30-APC, eBioScience), GPIbα (1C2-PE, BioLegend), GPVI (784808-AF647, R&D), CLEC-2 (17D9-PE, BioLegend), Ter119 (Ter119-AF488, BioLegend), CD62P (RMP-1-PE, BioLegend), and CD71 (C2F2-PE, BioLegend). For intracellular staining, cells were permeabilized using PBS–Triton X-100 (0.1%, Abcam) for 10 min, before staining for GATA-1, anti-TGF-β1-APC (TW7-16B4, BioLegend), and FAM-FLICA Caspase-1 assay kit (Immunochemistry Technologies). Cells were fixed in 4% formalin, before acquisition using an Accuri C6 flow cytometer (BD Bioscience, UK), and analyzed using FlowJo v10. In zymosan-induced peritonitis, peritoneal cells (all) and splenocytes (⅕) were aliquoted into 12 × 75 mm polystyrene FACS tubes and incubated 1:50 with mouse FcR block (130-092-575; Miltenyi Biotec) for 2 min at RT. Peritoneal cells and splenocytes were then stained with the following antibodies for 20 min at 4°C prior to washing and fixation with 2% PFA: anti-CD45.2 BV605 (clone 104; BioLegend), anti-CD11c PE-Cy7 (clone N418, ThermoFisher), anti-Siglec F TexasRed (clone E50-2440, BD), anti-Ly6C FITC (clone HK1.4, BioLegend), anti-Ly6G APC (clone 1A8, BD), and anti-F4/80 APC-eFluor780 (clone BM8, ThermoFisher). Splenocytes were also stained with anti-CD3 PECy7 (clone 145-2C11, ThermoFisher) and anti-CD19 APC (clone 1D3, ThermoFisher). Immediately prior to analysis, CountBright beads (Invitrogen) and Zombie Aqua (BioLegend) were added and samples were acquired using Fortessa-X20 and data analyzed offline using FlowJo (V-10.2.6).

Cells and CountBright beads were gated on using the forward (FSC) and side (SSC) scatter profiles. Doublets and dead cells were excluded using forward scatter area versus height (FSC-A and FSC-H), and positive zombie aqua signal, respectively. Leukocytes were identified based on positive expression of CD45, and subdivided into dendritic cells (CD11c^+^), eosinophils (SiglecF^+^), monocytes (Ly6C^+^), neutrophils (Ly6G^+^), macrophages (F4/80^hi^), T cells (CD3^+^), and B cells (CD19^+^). The absolute numbers of each leukocyte population listed above were quantified using the CountBright beads (ThermoFisher) according to the manufacturer’s instructions, and the obtained value was multiplied by the denominator of the fraction of tissue stained to provide the total number of leukocytes within that tissue.

### 2.8 Western blotting

Washed mouse platelets were isolated at 4 × 10^8^/ml, mixed with 2× Laemmli Sample Buffer (Bio-Rad, USA), and boiled. Protein electrophoresis was performed in Tris-Glycine SDS running buffer and transferred to an Immuno-Blot PVDF Membrane (Bio-Rad, USA). Membranes were imaged for peroxidase post-ECL substrate incubation using a LI-COR Odyssey Fc Imager and quantified using ImageJ.

### 2.9 Zymosan-induced peritonitis

Peritonitis was induced by intraperitoneal (IP) injection of 0.1 mg zymosan A (Z4250-250mg; Merck, UK) from *Saccharomyces cerevisiae*. After 4 h, mice were sacrificed *via* CO_2_ narcosis and cervical dislocation. The peritoneum was lavaged by injecting 5 ml of 5 mM EDTA (Sigma-Aldrich) diluted in PBS without Mg/Ca into the peritoneal cavity and massaging the peritoneal membrane to detach adherent cells. The peritoneal lavage fluid (PLF) was collected using a 19 G 2-inch needle to a 5-ml syringe and stored on ice. The spleen was collected into a 15- ml falcon tube containing PBS with Mg^2+^/Ca^2+^ and transported on ice. Tissues were processed immediately as described below.

### 2.10 Tissue processing

PLF samples were centrifuged at 300 g for 5 min to pellet cells. PLF supernatants were collected and stored at −80°C for future analysis. Peritoneal cells were then resuspended in MACS buffer [0.1 mM ethylenediaminetetraacetic acid (EDTA) and 0.6% bovine serum albumin (BSA) in PBS without Mg^2+^/Ca^2+^; all Sigma-Aldrich] and stored on ice prior to analysis by flow cytometry. Spleens were weighed using Pioneer weighing scales (Ohaus, Switzerland) and placed onto 40-µm cell strainers (Grenier Bio-One, Austria). Using the plunger of a 5-ml syringe, the spleen was mashed and washed through the cell strainer into the collection tube with PBS with Mg/Ca. Cells were pelleted by centrifugation at 300 g for 5 min. To lyse red blood cells (RBCs), 3 ml of formulated RBC lysis buffer (155 mM NH_4_Cl, 12 mM NaHCO_3_, and 0.1 mM EDTA; all from Sigma-Aldrich) was added to spleen samples for 10 min at RT. Splenocytes were then washed twice by diluting cells with 10 ml of PBS with Mg^2+^/Ca^2+^ followed by centrifugation at 300 g for 5 min. Splenocytes were then resuspended in MACS buffer and stored on ice prior to analysis by flow cytometry.

### 2.11 ELISA

Cytokines were measured in non-diluted PLF using Peprotech (UK) mini-ELISA kits according to the manufacturer’s instructions.

### 2.12 Peripheral blood smear staining

Blood smears were air-dried, fixed in methanol for 10 min, and stained with May–Grünwald Giemsa staining kit (Atom Scientific, UK) according to the manufacturer’s instructions.

### 2.13 Staining of bones for fibrosis

Femoral bones were fixed in 4% paraformaldehyde, decalcified, and embedded in paraffin. Sections were stained with an Elastic Van Gieson Stain Kit (Verhoeffs) (Atom Scientific UK) according to the manufacturer’s instructions.

### 2.14 Statistics

Data are presented as mean ± SD. For all peritonitis results, bar in dot plot represents median. The statistical difference between multiple groups was assessed by Kruskal–Wallis test or (for all peritonitis results) by one-way ANOVA or (for ELISA data and other comparison between two groups) by Mann–Whitney test using Prism 8 (GraphPad Software Inc, USA). Statistical significance is represented by asterisks: *p < 0.05, **p < 0.01, ***p < 0.001, ****p < 0.0001.

## 3 Results

### 3.1 *Nlrp3* ^A350V/+^/*Gp1ba-*Cre^KI/+^ mice have elevated levels of caspase-1 in platelets

**Figure 1:**
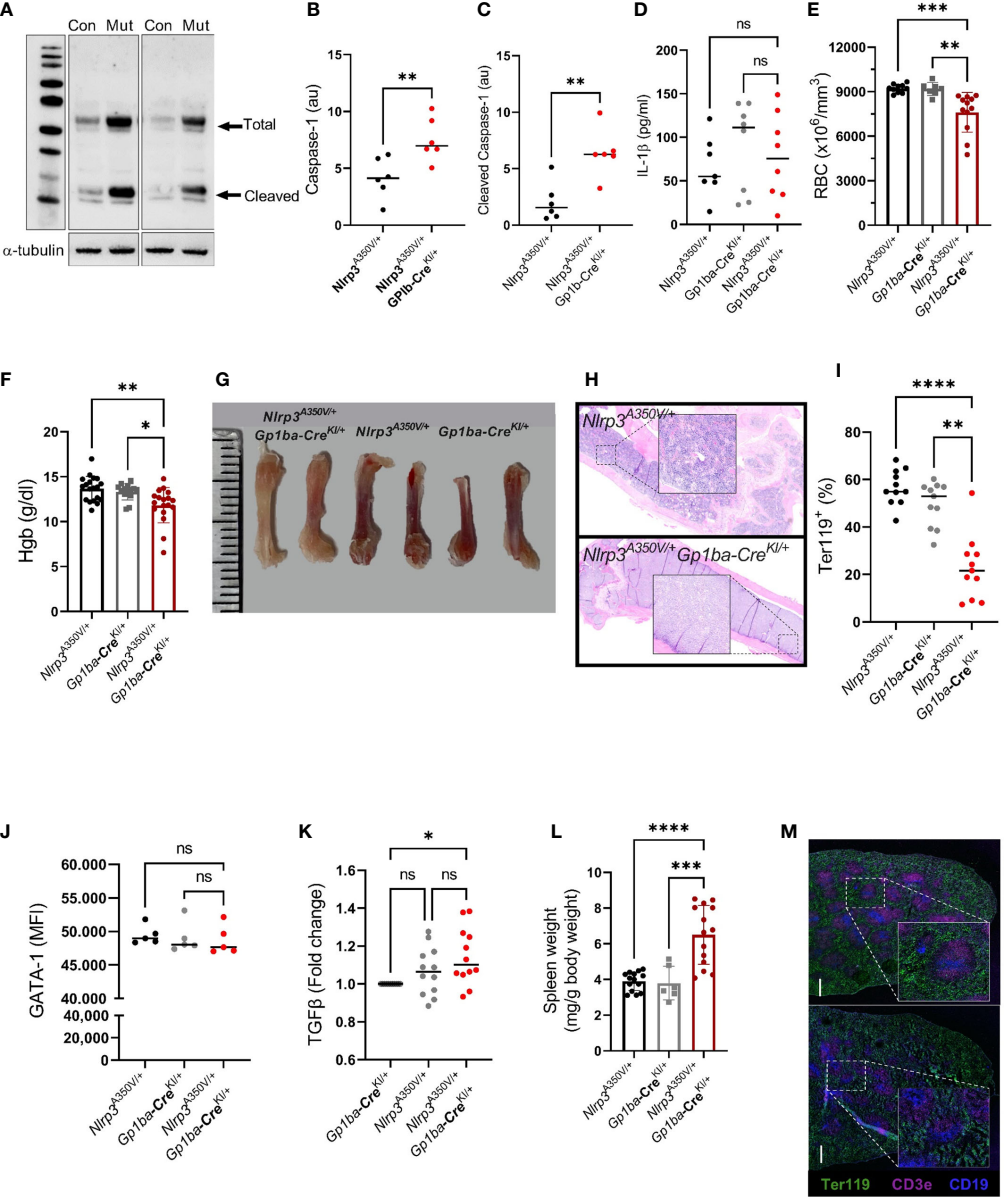
*Nlrp3* ^A350V/+^/*Gp1ba-*Cre^KI/+^ mice have elevated levels of caspase-1 and anemia. **(A)** Increased levels of both pro-caspase-1 and active enzyme in platelets from *Nlrp3* ^A350V/+^/*Gp1ba-*Cre^KI/+^ mice (Mut) compared to control (Con) platelets. Representative blots out of two, each containing three different control and three mutant mice. **(B, C)** Densitometry of total **(B)** and cleaved/active caspase-1 **(C)**, normalized to α-tubulin. **(D)** Plasma levels of IL-1β in *Nlrp3* ^A350V/+^/*Gp1ba-*Cre^KI/+^ vs. control mice; *n* = 7–8. **(E)** RBC counts and **(F)** hemoglobin contents in control vs. mutant mice, *n* = 7–19. **(G)** Representative femur bones from *Nlrp3* ^A350V/+^/*Gp1ba-*Cre^KI/+^ mice vs. control mice. Note the reduced red color in the bones of mutant animals. **(H)** Hematoxylin–eosin staining of femur bones of control (upper panel) vs. mutant (lower panel) mouse. Note the lower numbers of nucleated cells in the mutant BM. Representative images out of 3 mice per group. **(I)** Strongly reduced Ter119+ (erythroid lineage) cells in the BM of mutant mice. **(J)** GATA-1 levels (MFI) in erythroid (Ter119+) cells in the BM of mutant vs. control mice, *n* = 5 for each group. **(K)** Fold change of the TGFβ content in MKs from the BM of control vs. *Nlrp3* ^A350V/+^/*Gp1ba-*Cre^KI/+^ mice, *n* = 12. **(L)** Spleen weight normalized to the body weight, *n* = 7–14. **(M)** Staining of spleen sections of control (upper panel) vs. *Nlrp3* ^A350V/+^/*Gp1b*α−Cre^+/KI^ (lower panel) mice for Ter119 (pan-erythroid marker, green), CD3e (T-cell marker, pink), and CD19 (B-cell marker, blue). Representative sections out of three mice per group; ns, not significant.

MK/platelet-specific NLRP3 gain-of-function mice were generated by crossing *Nlrp3* ^A350V/+^-floxed (fl) mice with the *Gp1ba*-Cre knockin (KI) mice. The *Gp1ba*-Cre transgene was used rather than Pf4-Cre because it has been shown to be more lineage-specific (9), eliminating confounding effects of hyperactivation of NLRP3 in immune cells. *Nlrp3* ^A350V/+^/*Gp1ba-*Cre^KI/+^ mice developed normally (**Supplementary Figures 1A, B**) and did not demonstrate any visible abnormalities. First, we confirmed that the mutation results in increased activity of NLRP3 in cells of megakaryocytic origin. As activation of caspase-1 is one of the central outcomes of NLRP3 inflammasome function, we determined both pro-caspase-1 and the active protein in platelets using Western blotting. The intraplatelet level of both total and active caspase-1 was significantly higher in platelets from NLRP3 gain-of-function mice compared with controls (**Figures 1A–C**).

A variety of potential effects of NLRP3 hyperactivation could potentially be attributed to elevated plasma levels of active IL-1β secreted from platelets, especially given their abundance in the circulation. We therefore measured IL-1β concentration in plasma of both mutant and control mice. Surprisingly, the levels of the cytokine were similar in both control and mutant strains (**Figure 1D**). This could be based on the recently reported novel mechanism of IL-1β secretion from platelets, which is independent of inflammasome and caspases (14). Also, we used unchallenged mice and therefore these results cannot rule out potential participation of platelet-derived IL-1β in the presence of a challenge, for example, after platelet stimulation with LPS *in vitro* or following cecal ligation and puncture *in vivo* (15). Of note, the seeming difference between medians in the control groups was highly insignificant (*p* > 0.65 by ANOVA Kruskal–Wallis test).

Thus, any phenotypes observed in unchallenged *Nlrp3* ^A350V/+^/*Gp1ba-*Cre^KI/+^ mice would be unrelated to systemic alterations in the IL-1β level.

### 3.2 *Nlrp3* ^A350V/+^/*Gp1ba-*Cre^KI/+^ mice have mild anemia and reduced erythropoiesis in the bone marrow

Mutant mice had mildly decreased RBC counts (7,605 ± 1,340 vs. 9,214 ± 289 and 9,184 ± 430 × 10^3^/μl in controls, respectively) and hemoglobin (11.8 ± 1.9 g/dl vs. 13.7 ± 1.4 g/dl and 13.3 ± 0.9 g/dl in controls, respectively; **Figures 1E, F**), whereas the numbers of white blood cells remained unchanged (8.1 ± 4.3 vs. 5.2 ± 2.6 × 10^3^/μl in control, *p* > 2.3). To explore the cause of the anemia, we assessed erythropoiesis in the BM of the mutant mice. Femoral bones of the mutant mice had visually reduced red color (**Figure 1G**) and lower numbers of nucleated cells (**Figure 1H**). Consistent with this finding, the proportion of cells positive for the erythroid marker Ter119 was dramatically decreased in the *Nlrp3* ^A350V/+^/*Gp1ba-*Cre^KI/+^ mice (**Figure 1I**). In contrast, the number of MK did not differ between the mouse strains (**Supplementary Figure 1C**). These results imply that the numbers of the maturing erythroblasts are markedly reduced. No difference in BM fibrosis was observed (**Supplementary Figure 1D**). The percentage of reticulocytes (Ter119^+^CD71^+^ or Ter119^+^thiazole orange^+^, 10.9 ± 3.4 vs. 10.7 ± 0.7 and 11 ± 5.1 vs. 11.7 ± 1.8 in mutant and controls, respectively; *p* > 0.7) and young platelets (CD41^+^thiazole orange^+^, 10.8 ± 5.3 vs. 10.7 ± 0.8 in mutant and controls, respectively; *p* > 0.4) in the peripheral blood were similar in the mutant animals and controls. Peripheral blood smears of *Nlrp3* ^A350V/+^/*Gp1ba-*Cre^KI/+^ mice did not visually differ from controls (**Supplementary Figure 1E**).

### 3.3 Hyperactive MK NLRP3 could modulate erythropoiesis through TGF-β1 signaling

GATA1 is one of the major transcription factors supporting survival, proliferation, and differentiation of erythroid progenitors, downstream of the erythropoietin receptor (16). Thus, to investigate potential mechanisms of reduced RBC counts in *Nlrp3* ^A350V/+^/*Gp1ba-*Cre^KI/+^ mice, we measured levels of GATA1 in cells of erythroid lineage (Ter119^+^) in the BM of the mutant mice; however, no differences compared with control mice were observed (**Figure 1J**). A role of MK-derived TGF-β1 in the transition of erythroid progenitors to the erythropoietin-sensitive stage has recently been reported (17). Reduced numbers of Ter119^+^ precursors in the BM suggested that this mechanism could be implicated in the observed phenotype. Indeed, TGF-β1 levels were increased in the BM MK, although compared with only one (*Nlrp3* ^+/+^/*Gp1ba-*Cre^KI/+^) but not another (*Nlrp3* ^A350V/+^/*Gp1ba-*Cre^+/+^) control (**Figure 1K**). It is also known that CD109, a TGF-β coreceptor, downregulates TGF-β signaling in hematopoietic progenitors (18), and its modified expression could therefore mediate reduced maturation of the early precursors even in the absence or minimal changes in the levels of TGF-β itself.

Different subtypes of MK have recently been described, including platelet-forming, immunoregulatory, niche-supporting, and endomitotic cells (19). As immunoregulatory MKs are enriched with inflammatory markers and genes implicated in cytokine synthesis and secretion, it is tempting to hypothesize that the erythropoiesis-modifying stimulus, regulated by NLRP3, originates from this MK subtype. The percentage of this MK subtype, recognized as CD41^+^ cells expressing S100A9, was similar in mutants and controls (7.7 ± 4.2 vs. 5.8 ± 2.1, respectively, *p* > 0.6), which, however, does not rule out the potential difference in their cytokine production activity.

These findings reveal a novel and unexpected role of MK/platelet NLRP3 in erythropoiesis. Understanding the molecular mechanism underlying this phenomenon, in particular, whether NLRP3 operates alone or as a part of inflammasome, requires further investigation.

### 3.4 Erythropoiesis is expanded in the spleen of *Nlrp3* ^A350V/+^/*Gp1ba-*Cre^KI/+^ mice

The spleen was enlarged in the mutant mice, with its weight (normalized to the body weight) exceeding the weight of the spleen of control animals by 1.5-fold (6.7 ± 1.7 vs. 3.8 ± 0.6 and 3.9 ± 0.8 g in controls, respectively; **Figure 1L**). Staining of spleen sections revealed vague borders between the red and white pulp, which could reflect progressive growth of spleen size, but no prominent visible differences in the content of Ter119^+^ erythroid progenitors, and T or B cells, between control and *Nlrp3* ^A350V/+^/*Gp1ba-*Cre^KI/+^ mice (**Figure 1M**). This was further confirmed by flow cytometry, which showed similar proportion of Ter119^+^ cells and erythroblast subtypes, EryA, EryB, and EryC, as well as MKs in the spleen of mutant and control animals (**Supplementary Figures 1F–H**). Thus, the overall content of maturing erythroid precursors in the spleen is increased proportionally to its size, which likely reflects a compensatory effect for reduced BM erythropoiesis. Hyperactive NLRP3 in MK might create an inflammatory environment, which is known to suppress steady-state erythropoietin-induced BM erythropoiesis and simultaneously stimulate extramedullary stress erythropoiesis, in particular, in spleen (20). This mechanism could contribute to the observed phenotype.

### 3.5 *Nlrp3* ^A350V/+^/*Gp1ba-*Cre^KI/+^ mutation does not affect platelet function

As the NLRP3 activity in our mouse model is platelet-specific, we next assessed whether it affects key platelet functions. No difference in the platelet counts or expression of major platelet receptors, GPIbα, the integrin β3 subunit (CD41), CLEC-2, and GPVI was detected (**Supplementary Figures 2A–E**). The release of α-granules (as judged by surface P-selectin expression) was comparable in mutant vs. control platelets in response to either CRP or thrombin (**Supplementary Figures 2F, G**) as well as aggregation induced by CRP, thrombin, U46619 (a thromboxane A agonist), and H3 (**Supplementary Figures 2H–K**). It was previously reported that inhibition of the NLRP3 inflammasome through Btk kinase attenuated platelet activation, aggregation, and thrombus development *in vitro* (21). The discrepancy with our findings may be explained by the fact that genetic upregulation of NLRP3 activity in a lineage-specific manner may not necessarily produce an effect opposite to its inhibition. Moreover, potential additional downstream and off-target effects of Btk kinase inhibitors unrelated to the inflammasome cannot be ruled out.

No changes in platelet deposition on collagen surface at arterial shear rate was observed (**Supplementary Figures 2L–N**). Hyperactivity of NLRP3 in platelets also did not modify either thrombosis prevalence or thrombus size in the model of venous thrombosis induced by partial blood flow restriction in the IVC (**Supplementary Figures 2O–Q**). The latter has been shown to depend on platelets, as platelet depletion prevents thrombosis in the model (22).

### 3.6 *Nlrp3* ^A350V/+^/*Gp1ba-*Cre^KI/+^ mice have increased neutrophil influx and cytokine levels in the peritoneum under inflammatory conditions

**Figure 2:**
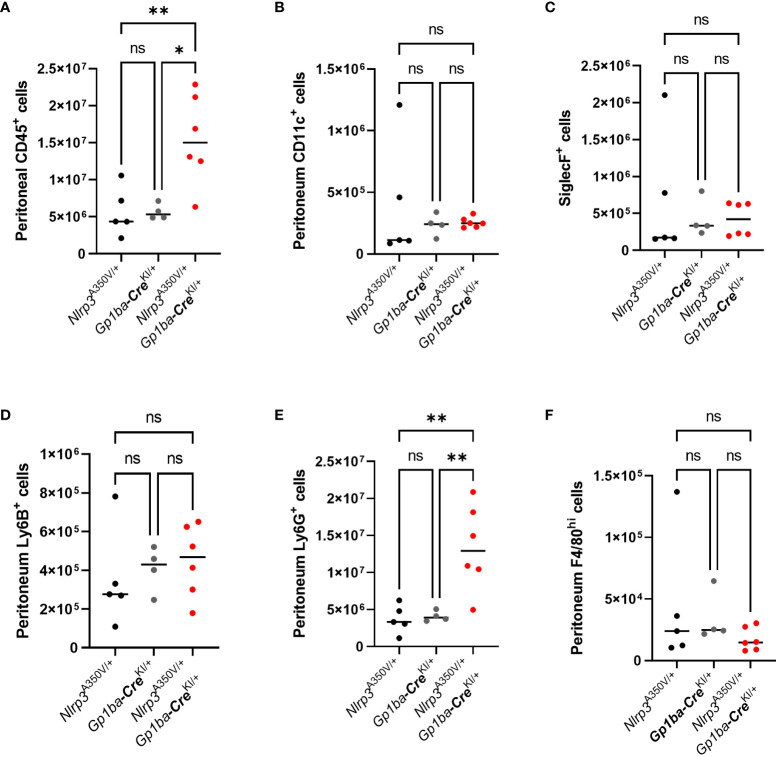
Increased accumulation of neutrophils in the peritoneal fluid of *Nlrp3* ^A350V/+^/*Gp1ba-Cre^KI/+^* mice in zymosan-induced peritonitis. Control and mutant mice were injected i.p. with zymosan, and in 4 h, PLF was collected. The following cells were counted by flow cytometry: CD45+ leukocytes **(A)**, CD11c^+^ dendritic cells **(B)**, SiglecF^+^ eosinophils **(C)**, Ly6B^+^ inflammatory monocytes **(D)**, Ly6G^+^ neutrophils **(E)**, and F4/80^+^ macrophages **(F)**. Bar represents median; *n* (control) = 4–5, *n* (*Nlrp3* ^A350V/+^/*Gp1ba-*Cre^KI/+^) = 6.

**Figure 3:**
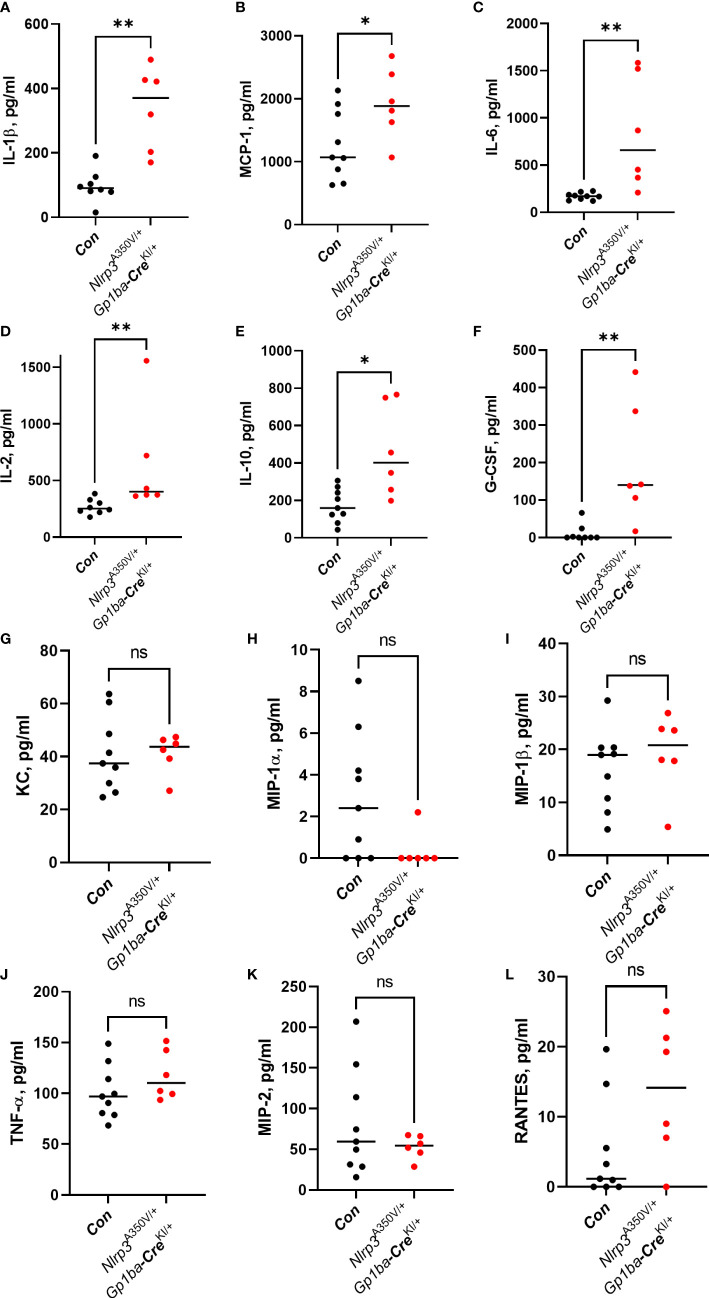
Cytokine levels in the PLF in zymosan-induced peritonitis. Levels of the following cytokines: IL-1β **(A)**, MCP-1 **(B)**, IL-6 **(C)**, IL-2 **(D)**, IL-10 **(E)**, G-CSF **(F)**, KC **(G)**, MIP-1α **(H)**, MIP-1β **(I)**, TNF-α **(J)**, MIP-2 **(K)**, and RANTES **(L)** were measured in the PLF 4 h after zymosan administration using commercial ELISA kits in accordance with the manufacturer’s instructions. Bar represents median; *n* (control, *Nlrp3* ^+/+^/*Gp1ba-*Cre^KI/+^ and *Nlrp3* ^A350V/+^/*Gp1ba-*Cre^+/+^ combined) = 9, *n* (*Nlrp3* ^A350V/+^/*Gp1ba-*Cre^KI/+^) = 6.

To assess whether overexpression of Nlrp3 in the MK lineage plays a role in the setting of inflammation, we used peritonitis induced by intraperitoneal injection of zymosan. *Nlrp3* ^A350V/+^/*Gp1ba-*Cre^KI/+^ mice had a robust influx of CD45^+^ leukocytes consisting of Ly6G^+^ neutrophils into the peritoneum, whereas the numbers of other cell types, such as CD11c^+^ dendritic cells, SiglecF^+^ eosinophils, Ly6B^+^ inflammatory monocytes, and F4/80^+^ macrophages, remained unchanged (**Figure 2**). No difference in immune cell numbers in spleen was observed (**Supplementary Figure 3**). The levels of a set of proinflammatory cytokines, including IL-1β, MCP-1, IL-6, and IL-2, were also elevated in PLF (**Figures 3A–D**). These findings imply that MK/platelet NLRP3 is one of the mediators of acute inflammation and corroborates the observation that IL-1β-deficient mice have reduced inflammatory response in zymosan-induced peritonitis (23). Interestingly, the mutant mice had also increased levels of a potent anti-inflammatory cytokine, IL-10 (**Figure 3E**), which inhibits Nlrp3 inflammasome and synthesis of IL-1β (24). The levels of G-CSF, a major factor stimulating production of neutrophils, were also increased (**Figure 3F**). This is in line with the reported ability of IL-1β to stimulate G-CSF production (25), whereas G-CSF, in turn, can upregulate IL-1β secretion (26). In addition, G-CSF blocks erythropoiesis in the BM (27), which could potentially worsen anemia in the context of MK/platelet NLRP3 hyperactivation. Surprisingly, keratinocyte chemoattractant (KC, CXCL1), a potent chemoattractant for neutrophils, was not increased (**Figure 3G**). Thus, the observed neutrophil egression is mediated by other chemokines. For example, MCP-1 can promote neutrophil recruitment (28). However, KC levels could have already returned to control levels at 4 h as its peak at 2 h has been previously reported (29). Levels of MIP-1α, MIP-1β, TNF-α, MIP-2, and RANTES also remained unchanged (**Figures 3H–L**). Thus, hyperactivity of NLRP3 in MK and platelets results in a robust increase in the inflammatory response in the peritonitis setting.

## 4 Discussion

In this study, we used a mouse model that expresses hyperactive NLRP3 specifically in the MK lineage. As expected, increased levels of an active form of caspase-1 were detected in platelets of these mice. However, surprisingly, total caspase-1 (the enzyme precursor) was also elevated. The reason for this is incompletely clear although one of the possible mechanisms is that increased production of IL-1β in the BM, resulting from upregulated activity of MK NLRP3, stimulates NF-κB (30) in MK leading to enhanced synthesis of caspase-1 proenzyme (31), which is later found in circulating platelets.

Using these mice, we have demonstrated the specific biological role of the NLRP3 inflammasome associated with cells of the MK lineage, in erythropoiesis and inflammation. In unchallenged mice, hyperactivity of MK/platelet NLRP3 resulted in mild anemia, surprisingly, without affecting platelet numbers or function. Marked reduction of erythropoiesis in the BM of mutant mice was accompanied by its shift to the spleen, a potential compensatory mechanism. Mechanisms of MK NLRP3 involvement in erythropoiesis require further investigation although one possible candidate could be MK-derived TGF-β1. Indeed, in a recent study, MK-generated TGF-β1 was shown to couple pre-erythropoietin-dependent stage erythropoiesis to its late erythropoietin-dependent stage (17). In this study, however, the level of TGF-β1 in the BM was moderately increased and therefore these findings cannot explain the observed anemia. Equally, it cannot be attributed to TGF-β1-related BM fibrosis as we did not observe it in the mutant mice. Conversely, TGF-β superfamily traps, which prevent ligand/receptor interaction and inhibit downstream signaling, have been shown to boost erythropoiesis increasing hemoglobin, hematocrit, and RBC counts (32, 33). Although the mechanism by which TGF-β superfamily traps stimulate erythropoiesis is incompletely understood, we can speculate that the increase in BM TGF-β1 could inhibit RBC production, which could mediate, at least in part, the mild anemia observed in the mutant mice.

Interestingly, a gain-of-function mutation in another inflammasome, NLRP1a, stimulated pyroptosis of hematopoietic progenitor cells (34). Hyperactivity of NLRP3 did not change the systemic levels of IL-1β, which suggests that suppression of erythropoiesis in the BM is likely a local paracrine effect. As NLRP3 inflammasome is involved in the activation of two cytokines, IL-1β and/or IL-18, the observed effect could be attributed to either of them, or both. For example, it has been reported that IL-1β exposure reduced hematopoietic cell renewal both directly and *via* BM niche cells (35, 36). Moreover, from a set of inflammatory cytokines, IL-1β has been shown to be the strongest inhibitor of hypoxia-induced erythropoietin production *in vitro* (37). Moreover, an association of IL-18 with reduced RBC production has also been reported (38). The relative contribution of IL-1β and/or IL-18 released by MK remains to be elucidated and warrants further investigation.

An acute inflammatory challenge induced by administration of zymosan resulted in a robustly enhanced response in the mutant animals, which included both a dramatic increase in neutrophil recruitment and the release of a variety of cytokines. This finding suggests that platelet-derived IL-1β and/or IL-18 play a key role in systemic inflammation. Active platelets have the entire splicing machinery, and are able to splice the endogenous pre-mRNA into a mature message and produce IL-1β protein similarly to nucleated cells (39). On the other hand, systemic inflammatory states, such as sepsis, are accompanied by platelet activation, which may simultaneously activate mRNA maturation and NLRP3 inflammasome assembly, thereby forming a full process of production and release of the active cytokines. Given high numbers of circulating platelets, which exceeds numbers of leukocytes by orders of magnitude, it is plausible that significant release of functional IL-1β and/or IL-18 could perpetuate the systemic inflammatory response to a similar degree as that observed with immune cells.

In conclusion, we have demonstrated for the first time a critical role of NLRP3 in cells of the MK lineage in RBC production and systemic inflammatory response. Targeting different components of the NLRP3 system in MK or (activated) platelets may be a promising tool to boost erythropoiesis or reduce inflammation.

## Data availability statement

The original contributions presented in the study are included in the article/**Supplementary Material**. Further inquiries can be directed to the corresponding author.

## Ethics statement

The animal study has been reviewed and approved by the local Ethics Committee of the University of Birmingham and the United Kingdom Home Office

## Author contributions

AB, JR, JC, JB, and AI designed the study; JC, JB, AB, SH, KW, and AI performed the experiments; AB, JR, JC, JB, KW, AI, YS, and JP interpreted the results; AB wrote the manuscript; JP, YS, JR, AI, SH, JC, and JB contributed to manuscript preparation and its critical discussion. All authors contributed to the article and approved the submitted version.

## Supplementary material

Supplementary Figure 1 Normal development and MK/erythroblast content in BM and spleen of *Nlrp3* ^A350V/+^/*Gp1ba-*Cre^KI/+^ mice. Body weight of *Nlrp3* ^A350V/+^/*Gp1ba-*Cre^KI/+^ male **(A)** and female **(B)** mice (red squares) as opposed to control animals (*NLRP^3^* ^A350V/+^/*Gp1ba-*Cre^+/+^, black triangles, and *NLRP^+^* ^/+^/*Gp1ba-*Cre^KI/+^, blue squares), *n* = 11 for all groups/sexes. **(C)** Percentage of MKs (CD41^+^ CD42b^+^ cells) in the BM of mutant vs. control animals. **(D)** Elastic van Gieson staining of control (left panel) and *Nlrp3* ^A350V/+^/*Gp1ba-*Cre^KI/+^ (right panel) femurs. Bone/connective tissue is pink, RBCs are light green, and cell nuclei are dark. Bar, 50 μm. **(E)** Smears of peripheral blood stained with May–Grünwald Giemsa, control (left panel) and *Nlrp3* ^A350V/+^/*Gp1ba-*Cre^KI/+^ (right panel). Bar, 20 μm. **(F)** Unaltered content of erythroid precursors at different stages of maturation in the spleen of mutant vs. control mice. **(G)** Percentage of MK and **(H)** Ter119+ erythroid progenitors in the spleen of *Nlrp3* ^A350V/+^/*Gp1ba-*Cre^KI/+^ mice vs. controls; *n* = 7–10.

Supplementary Figure 2 Normal platelet count, function, and thrombosis in *Nlrp3* ^A350V/+^/*Gp1ba-*Cre^KI/+^ mice. **(A)** Platelet counts in the mutant vs. control mice, *n* = 5–10. Unchanged expression of GPVI **(B)**, CD41/GPIIb-IIIa **(C)**, GPIbα **(D)**, and CLEC-2 **(E)** in *Nlrp3* ^A350V/+^/*GP1ba*Cre^+/KI^ mice compared to controls, *n* = 3. P-selectin expression on the surface of mutant vs. control platelets with or without activation by CRP **(F)** or thrombin **(G)**, *n* = 3. Representative aggregation curves and maximal aggregation amplitude of mutant vs. control platelets induced by CRP (10 µg/ml, **H**), thrombin (0.1 U/ml, **I**), U46619 (2.5 mM, **J**), or histone 3 (10 µg/ml, **K**), *n* = 3. **(L)** Deposition of platelets from control (left panel) and *Nlrp3* ^A350V/+^/*Gp1ba-*Cre^KI/+^ (right panel) mice on Horm collagen at 1,000 s^−1^. Representative images out of three independent experiments. **(M)** Surface coverage of mutant vs. control platelets adhered to collagen, representative data of one experiment with platelet deposition recorded during 4 min, *n* = 3 for each group. **(N)** Statistical comparison of platelet deposition on collagen, data from three independent experiments. MK/platelet-specific *Nlrp3* ^A350V/+^/*Gp1ba-*Cre^KI/+^ mutation does not alter venous thrombosis; thrombosis prevalence **(O)**, thrombus weight **(P)**, and thrombus length **(Q)** are presented, *n* = 8 for each group.

Supplementary Figure 3 Cell populations in spleen after zymosan-induced peritonitis. Populations of the following cells were analyzed in spleen 4 h after zymosan i.p. injection: CD45^+^ leukocytes **(A)**, CD11c^+^ dendritic cells **(B)**, SiglecF^+^ eosinophils **(C)**, Ly6B^+^ inflammatory monocytes **(D)**, Ly6G^+^ neutrophils **(E)**, and F4/80^+^ macrophages **(F)**, CD3^+^ T-cells **(G)**, and CD19^+^ B-cells **(H)**. Bar represents median; *n* (control) = 4–5, *n* (*Nlrp3* ^A350V/+^/*Gp1ba-*Cre^KI/+^) = 6.
