# Supplementary figures and images for: Megakaryocyte NLRP3 hyperactivation induces mild anemia and potentiates inflammatory response in mice
Source: Front Immunol. 2023 Aug 9;14:1226196. doi: 10.3389/fimmu.2023.1226196 (PMC10445124; doi:10.3389/fimmu.2023.1226196)

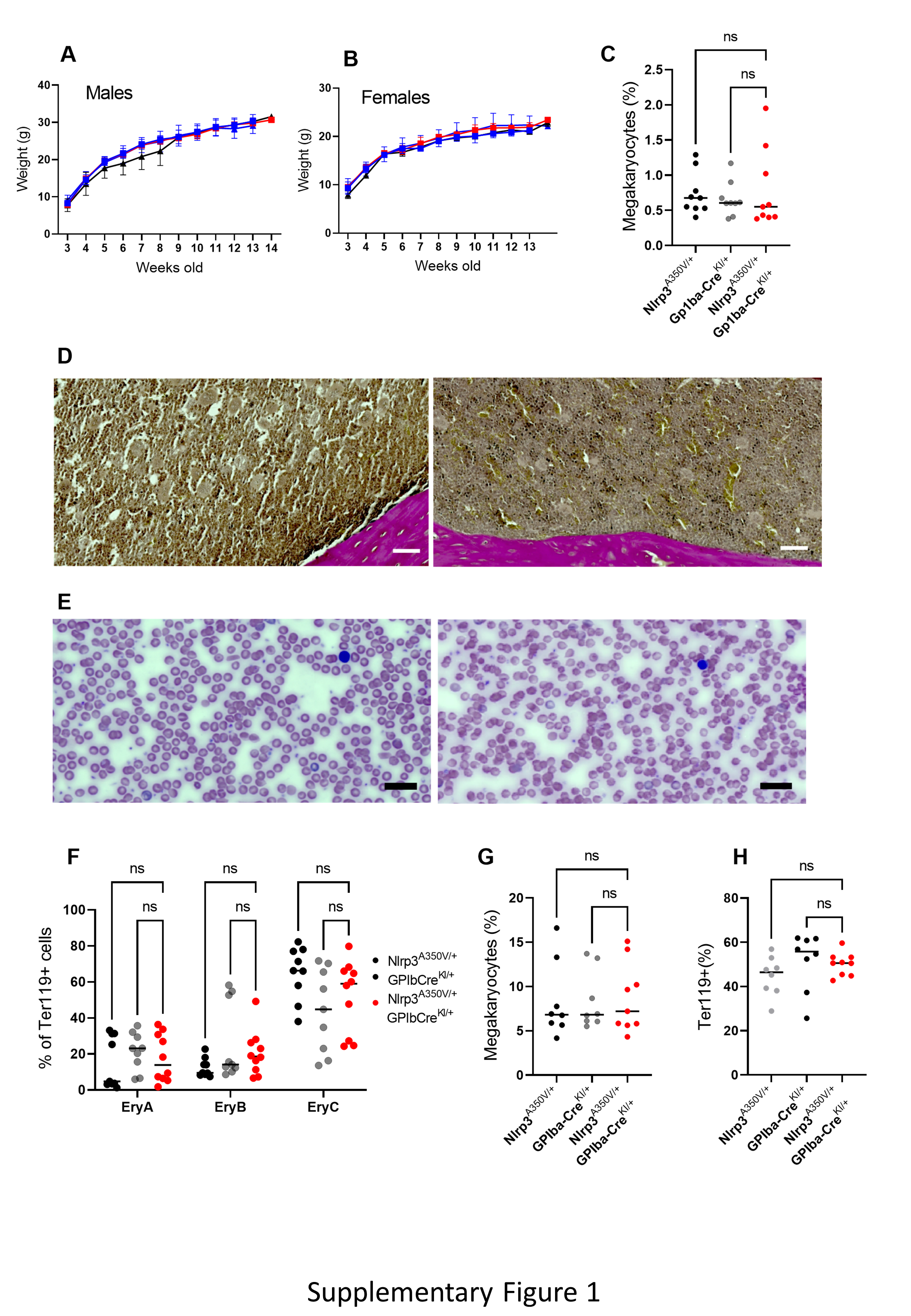

Supplement: Supplementary Figure 1 — Normal development and MK/erythroblast content in BM and spleen of Nlrp3 A350V/+/Gp1ba-CreKI/+ mice. Body weight of Nlrp3 A350V/+/Gp1ba-CreKI/+ male (A) and female (B) mice (red squares) as opposed to control animals (NLRP3 A350V/+/Gp1ba-Cre+/+, black triangles, and NLRP+ /+/Gp1ba-CreKI/+, blue squares), n = 11 for all groups/sexes. (C) Percentage of MKs (CD41+ CD42b+ cells) in the BM of mutant vs. control animals. (D) Elastic van Gieson staining of control (left panel) and Nlrp3 A350V/+/Gp1ba-CreKI/+ (right panel) femurs. Bone/connective tissue is pink, RBCs are light green, and cell nuclei are dark. Bar, 50 μm. (E) Smears of peripheral blood stained with May–Grünwald Giemsa, control (left panel) and Nlrp3 A350V/+/Gp1ba-CreKI/+ (right panel). Bar, 20 μm. (F) Unaltered content of erythroid precursors at different stages of maturation in the spleen of mutant vs. control mice. (G) Percentage of MK and (H) Ter119+ erythroid progenitors in the spleen of Nlrp3 A350V/+/Gp1ba-CreKI/+ mice vs. controls; n = 7–10. [file Image_1.tif]

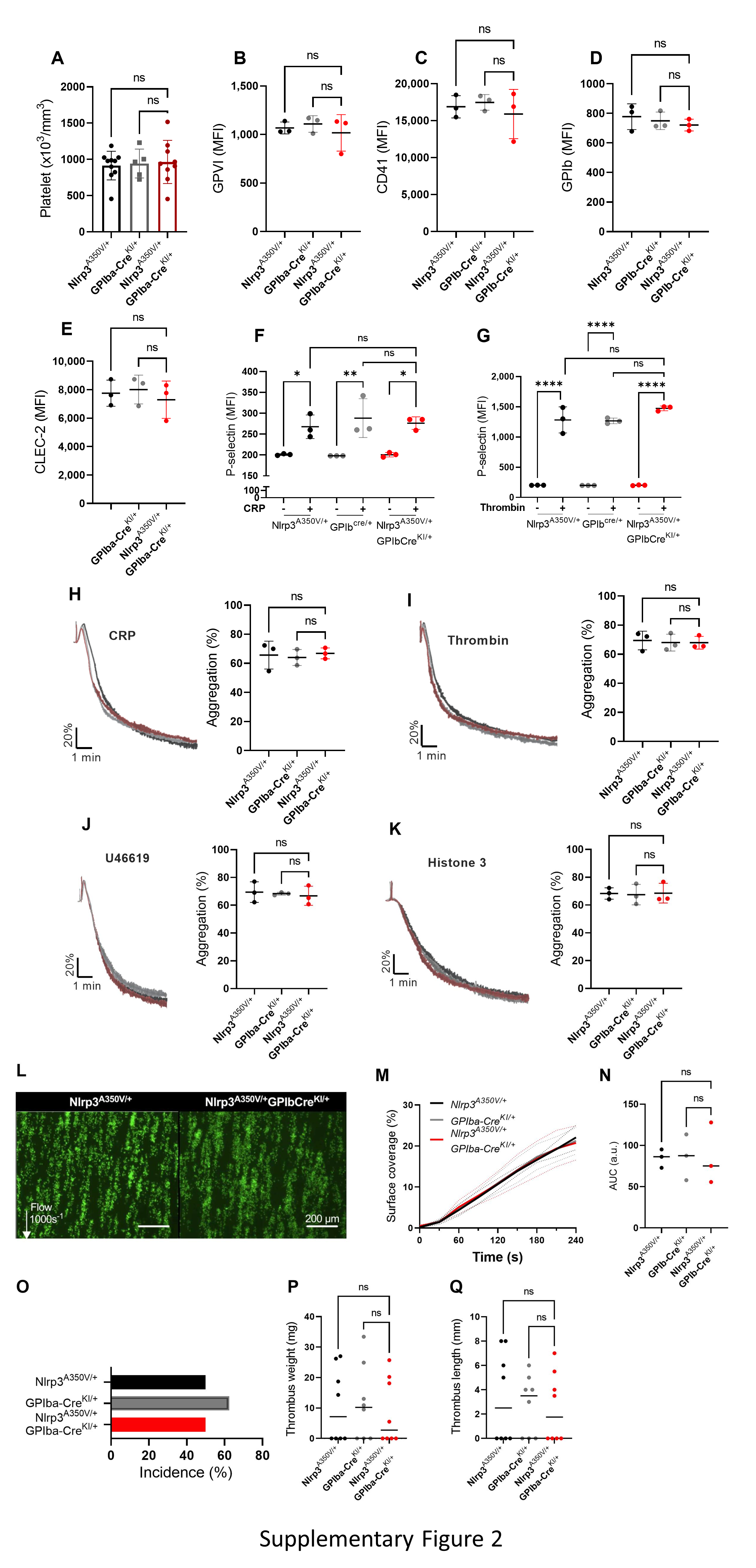

Supplement: Supplementary Figure 2 — Normal platelet count, function, and thrombosis in Nlrp3 A350V/+/Gp1ba-CreKI/+ mice. (A) Platelet counts in the mutant vs. control mice, n = 5–10. Unchanged expression of GPVI (B), CD41/GPIIb-IIIa (C), GPIbα (D), and CLEC-2 (E) in Nlrp3 A350V/+/GP1baCre+/KI mice compared to controls, n = 3. P-selectin expression on the surface of mutant vs. control platelets with or without activation by CRP (F) or thrombin (G), n = 3. Representative aggregation curves and maximal aggregation amplitude of mutant vs. control platelets induced by CRP (10 µg/ml, H), thrombin (0.1 U/ml, I), U46619 (2.5 mM, J), or histone 3 (10 µg/ml, K), n = 3. (L) Deposition of platelets from control (left panel) and Nlrp3 A350V/+/Gp1ba-CreKI/+ (right panel) mice on Horm collagen at 1,000 s−1. Representative images out of three independent experiments. (M) Surface coverage of mutant vs. control platelets adhered to collagen, representative data of one experiment with platelet deposition recorded during 4 min, n = 3 for each group. (N) Statistical comparison of platelet deposition on collagen, data from three independent experiments. MK/platelet-specific Nlrp3 A350V/+/Gp1ba-CreKI/+ mutation does not alter venous thrombosis; thrombosis prevalence (O), thrombus weight (P), and thrombus length (Q) are presented, n = 8 for each group. [file Image_2.tif]

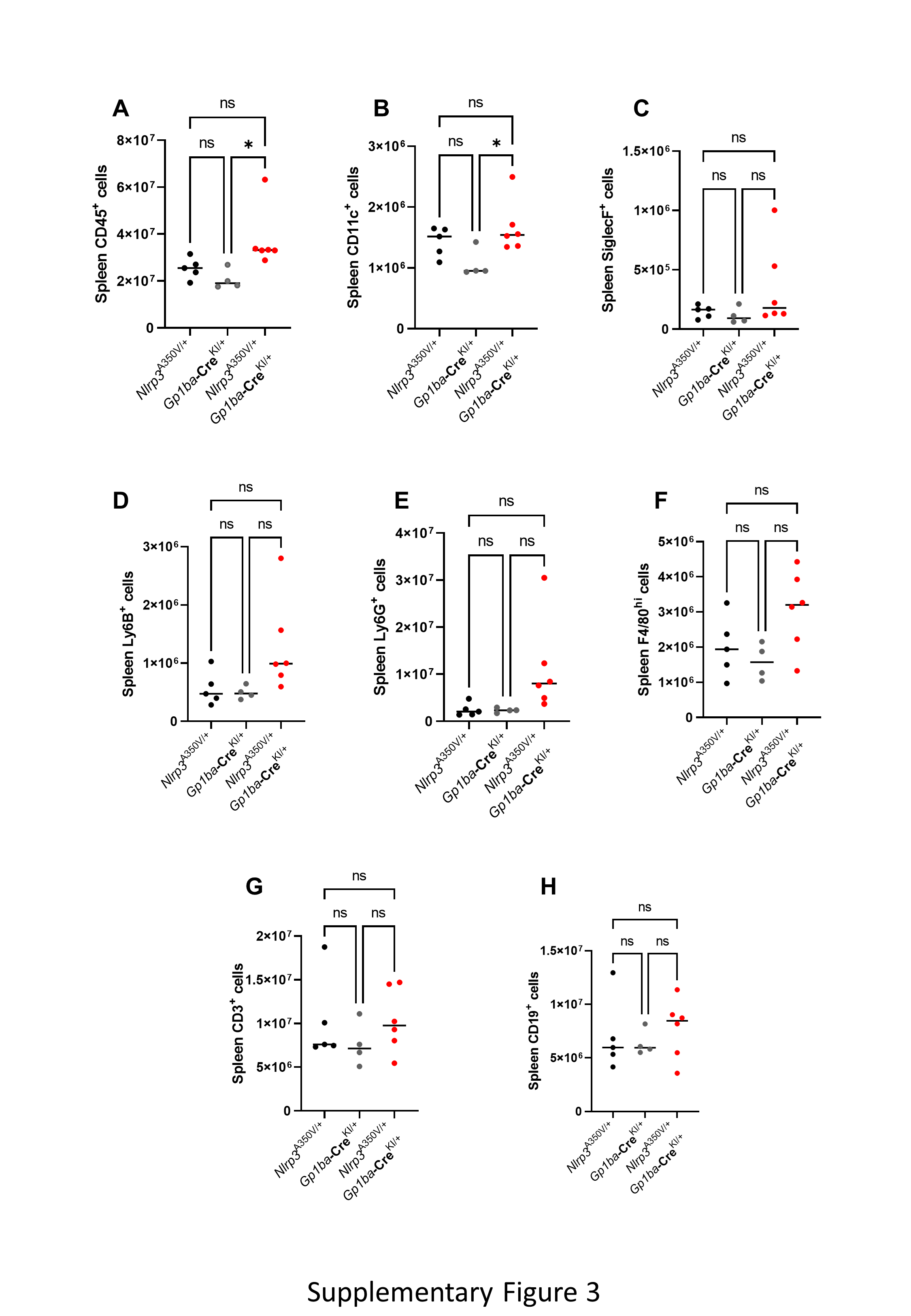

Supplement: Supplementary Figure 3 — Cell populations in spleen after zymosan-induced peritonitis. Populations of the following cells were analyzed in spleen 4 h after zymosan i.p. injection: CD45+ leukocytes (A), CD11c+ dendritic cells (B), SiglecF+ eosinophils (C), Ly6B+ inflammatory monocytes (D), Ly6G+ neutrophils (E), and F4/80+ macrophages (F), CD3+ T-cells (G), and CD19+ B-cells (H). Bar represents median; n (control) = 4–5, n (Nlrp3 A350V/+/Gp1ba-CreKI/+) = 6. [file Image_3.tif]
